# Gene Expression of Inflammatory Cytokines in Major Organs by Extracorporeal Circulation

**DOI:** 10.3390/jcm12082813

**Published:** 2023-04-11

**Authors:** Takuya Abe, Haruo Hanawa, Yutaka Fujii

**Affiliations:** 1 Department of Clinical Engineering and Medical Technology, Niigata University of Health and Welfare, Niigata 950-3198, Japan; 2 Department of Health and Sports, Niigata University of Health and Welfare, Niigata 950-3198, Japan

**Keywords:** extracorporeal circulation, rat ECC model, inflammatory response, biological reaction, real-time PCR

## Abstract

(1) Background: Extracorporeal circulation (ECC) is indispensable for cardiac surgery. Despite the fact that ECC causes non-physiological damage to blood components, its pathophysiology has not been fully elucidated. In our previous study, we constructed a rat ECC system and observed a systemic inflammatory response during and after blood tests assessing ECC, while the damage per organ localization caused by ECC was not examined. In this study, we used a rat model to assess the gene expression of inflammatory cytokines in major organs during ECC. (2) Methods: The ECC system consisted of a membranous oxygenator, tubing line, and a small roller pump. Rats were divided into a SHAM (which received surgical preparation only, without ECC) group and an ECC group. Proinflammatory cytokines were measured using real-time PCR in major organs after ECC to evaluate local inflammatory responses in the organs. (3) Results: Interleukin (IL)-6 levels were significantly elevated in the ECC group compared to the SHAM group, especially in the heart and lungs. (4) Conclusions: This study suggests that ECC promotes organ damage and the inflammatory response, but the degree of gene expression of proinflammatory cytokines varies from organ to organ, suggesting that it does not uniformly cause organ damage.

## 1. Introduction

In clinical practice, extracorporeal life support (ECLS), such as the cardiopulmonary bypass, is important because it keeps patients alive by ensuring adequate oxygen supply and blood flow to major organs [1]. However, cardiac surgical procedures using extracorporeal circulation (ECC) are often accompanied by a systemic inflammatory response that significantly affects post-ECC infection morbidity and mortality [2]. Further research is needed to elucidate the pathophysiological characteristics presented during ECC, but the difficulties of clinical and animal studies hamper such analyses. In our previous study, we established a small animal ECC model and evaluated systemic inflammatory responses in ECC [3]. The presence of systemic inflammation during ECC was supported; however, the damage that ECC can cause per organ localization was not examined. ECC causes a systemic reaction, and there are concerns regarding the occurrence of damage to the myocardium due to a cannula’s insertion into the right atrium and damage to the lungs due to reduced pulmonary blood flow [4]. It is important to study the mechanisms of pathophysiological changes during artificial blood perfusion.

In this study, we used a small animal model to assess the gene expression of inflammatory cytokines in major organs during ECC.

## 2. Materials and Methods

### 2.1. Animals

This study was approved by the Niigata University of Health and Welfare’s animal care and use committee, and all procedures complied with the National Institutes of Health’s guidelines for laboratory animal welfare. Additionally, 14–16-week-old Sprague–Dawley rats (male 400–450 g) were housed three rats per cage under a 12 h light–dark cycle with food and water available ad libitum. All animals were provided by CLEA Japan, Inc. (Tokyo, Japan).

### 2.2. Anesthesia, Surgical Preparation, and Extracorporeal Circulation

**Figure 1:**
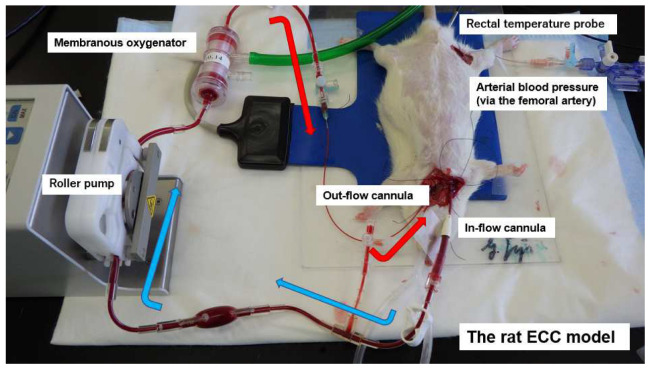
Extracorporeal circulation in a rat model.

The animals were anesthetized by isoflurane (4.5–5.0%) in oxygen-enriched air and placed in the supine position, with a rectal thermocouple probe in place. The rats were orotracheally intubated using a 14G cannula (Terumo Corp., Tokyo, Japan) and ventilated with a respirator (Model 687 respirator, Harvard Apparatus Ltd., Edenbridge, Kent, UK). Ventilation was volume-controlled at a frequency of 70–80 breaths/min, a tidal volume of 8–10 mL/kg body weight, and an inspired oxygen fraction of 30–40%. Anesthesia was maintained with 1.5–2.5% isoflurane (without neuromuscular blocking agents). Rectal temperature was maintained at 35.5–36.5 °C during the experiment. Arterial blood pressure was monitored (Model 870, PowerLab system, AD Instruments, Castle Hill, NSW, Australia) via the femoral artery, which was cannulated with polyethylene tubing (outside diameter: 0.8 mm; inner diameter: 0.5 mm; SP-31: Natsume Seisakusho Co., Ltd., Tokyo, Japan). The left common carotid artery was cannulated with polyethylene tubing (SP-55: outside diameter: 1.2 mm; inner diameter: 0.8 mm; SP-55: Natsume Seisakusho Co.) to serve as the arterial inflow cannula for the ECC circuit. Then, 500 IU/kg heparin sodium was administered after the placement of the arterial inflow cannula. A 16G 4-side hole cannula (depth: 38 mm; Togomedkit Co. Ltd., Tokyo, Japan) was advanced through the right external jugular vein into the right atrium and served as a conduit for venous outflow. The ECC system was composed of a polyvinyl chloride tubing circuit (Senko Medical Co., Ltd., Tokyo, Japan), a specially designed membranous oxygenator for small animals (polypropylene; membrane area: 0.039 m^2^; Senko Medical Co., Ltd., Osaka, Japan), and a small roller pump (REGLO Digital ISM831, ISMATEC, Wertheim, Germany) primed by 7 mL of saline and 1 mL (1000 IU) of heparin. The rat’s total blood volume was 8% of its body weight, and the dilution rate was approximately 25%. Figure 1 shows the experimental conditions.

### 2.3. Experimental Design

The animals were divided into two groups: the SHAM group (n = 4) and the ECC group (n = 3). The SHAM group received surgical preparation only, without ECC. The ECC pump flow was initiated and maintained at 60–70 mL/kg/min. The arterial pressure of carbon dioxide (PaCO_2_) and arterial pressure of oxygen (PaO_2_) were maintained at 35–45 mmHg and 300–400 mmHg, respectively, and managed with α-stat in the ECC group. Blood samples were collected at 3 defined time points: before ECC (pre-ECC) and 60 and 120 min after the initiation of ECC (end-ECC). All animals were sacrificed by myocardial potassium infusion at the end of ECC (120 min) weaning. Organ samples were harvested following euthanasia. They were collected from four organs: heart, liver, kidneys, and lungs. To evaluate local inflammatory responses, monocyte chemoattractant protein (MCP)-1, Interleukin (IL)-6, and tumor necrosis factor (TNF)-α levels were measured via real-time PCR. Blood gases, pH, hemoglobin (Hb) concentration, and electrolytes were also measured. Animals in which the Hb level declined to less than 7 g/dL at any point were excluded from the study.

### 2.4. Quantitative Reverse Transcription-Polymerase Chain Reaction

Small sections of the heart (left ventricle), liver, kidney, and lung (three places in each organ) were extracted to examine MCP-1, IL-6, and TNF-α expression. Total RNA was isolated from the organ tissue described above using Trizol (Life Technologies, Tokyo, Japan). cDNA was synthesized from 5 µg of total RNA with random primers. A plasmid containing rat MCP-1 cDNAs was used as the standard sample for the quantitative reverse transcription polymerase chain reaction of MCP-1. To construct the standard plasmid, cDNAs of MCP-1 were amplified using the primers 5′-ctgtctcagccagatgcagttaat-3′ and 5′-tatgggtcaagttcacattcaaag-3′ from rat heart, liver, kidney, and lung cDNA. The amplified cDNAs were directly inserted into the pGEM-T vector and the recombinant plasmids were purified. cDNA and diluted plasmid were amplified with the same primer used to create the plasmid and via LightCycler-FastStart DNA Master SYBR Green I (Roche, Indianapolis, IN) developed by LightCycler [5]. Using this plasmid standard curve, the absolute copy number of all samples (molecular mRNA/g of total RNA) was calculated using LightCycler software. In addition, to assess the mechanism of inflammation, the expressions of IL-6 and TNF-α mRNA were similarly examined using the following primers: IL-6—5′-aatttgcctattgaaaatctgctc-3′ and 5′ -ttcttcaagtgctttcaagatgag-3′; TNF-α—5′-atgggctccctctcatcagt-3′ and 5′-actccagctgctcctctgct-3′.

Additionally, γ-actin mRNA, acting as a housekeeping gene, was similarly examined using primers (gamma-actin—5′-agccttccttcctgggcatggagt-3′ and 5′-tggaggggcctgactcgtcatact-3′). The RNA level was adjusted according to the expression level of the housekeeping gene, and the expression level of the cytokine gene was quantified.

### 2.5. Real-Time PCR

MCP-1, IL-6, and TNF-α were analyzed using CFX Connect™ (BioRad Tokyo, Japan) using Gotaq^®^ qPCR Master Mix (promega, Tokyo, Japan) with the aforementioned primers. CFX Connect™ was initially denatured at 95 °C for 3 min, followed by 50 cycles of 3 steps of 95 °C for 10 s, 55 °C for 10 s, and 72 °C for 50 s.

### 2.6. Wet-to-Dry Ratio (Measurement of Indicators of Tissue Edema)

All animals were sacrificed at the end of ECC, and their left lungs were harvested and divided into three parts. The superior third was used for the calculation of the wet-to-dry (W/D) ratio. The lung block was weighed before and after desiccation for 48 h in a drying oven at 70 °C.

### 2.7. Statistics

All data are expressed as means ± standard error. Comparisons between groups were made by means of an unpaired Mann–Whitney U test. All statistical analyses were performed using IBM SPSS Statistics 27 (IBM SPSS, Chicago, IL, USA). Significance was set at *p* < 0.05.

## 3. Results

**Figure 2:**
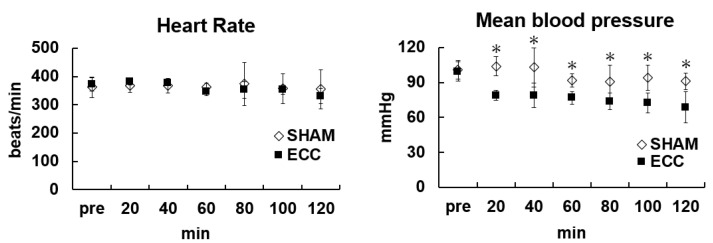
Hemodynamic changes (heart rate and mean blood pressure). * *p* < 0.05 versus SHAM group at the same time point.

The hemodynamics during the experiment in the SHAM and ECC groups are shown in Figure 2, and changes in Hb concentration, PaO_2_, PaCO_2_, and electrolyte levels are shown in Table 1. Mean arterial pressure (MAP) and Hb were significantly decreased during ECC. The PaO_2_ level was significantly higher (*p* < 0.05) in the ECC group than in the SHAM group. No statistical difference was found regarding the PaCO_2_ level between these groups.

**Figure 3:**
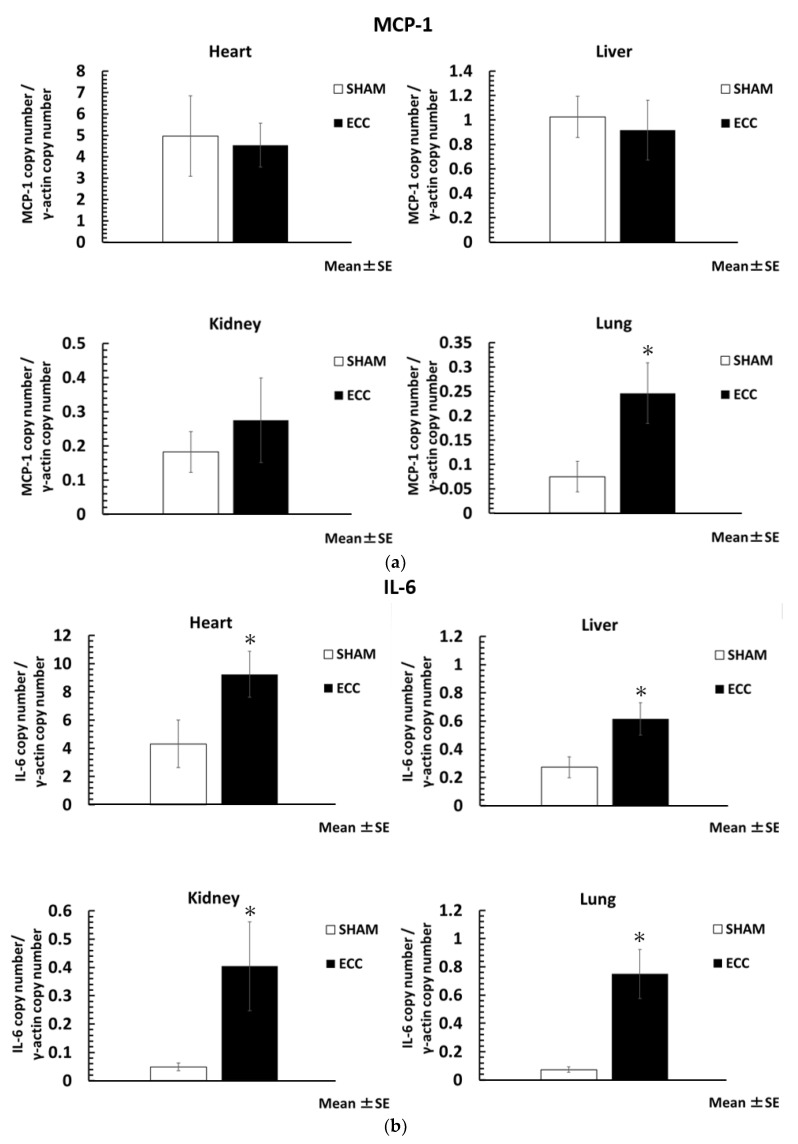

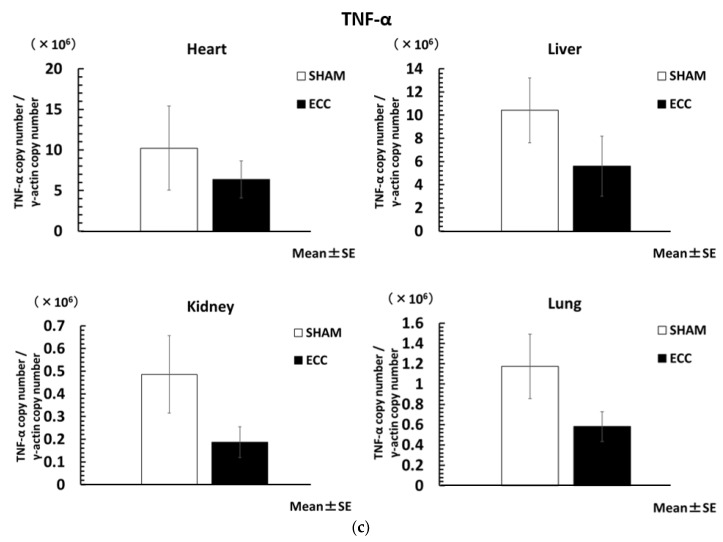
Monocyte chemoattractant protein (MCP)-1 (**a**), interleukin (IL)-6 (**b**), and tumor necrosis factor (TNF)-α (**c**); * *p* < 0.05 versus SHAM group.

The level of inflammatory cytokines was significantly increased in the ECC group compared to the SHAM group. The ratio of the housekeeping gene (γ-actin) to inflammatory cytokines is shown in Figure 3a–c. In particular, MCP-1 was significantly increased in the lungs (MCP-1/γ-actin; lungs: 0.25 ± 0.06), and IL-6 was significantly increased in all major organs (IL-6/γ-actin; heart: 9.25 ± 1.64: liver: 0.62 ± 0.12; kidney: 0.40 ± 0.16; lungs: 0.75 ± 0.17). In contrast, the gene expression of TNF-α was not increased at the organ level, nor was there a statistical difference.

**Figure 4:**
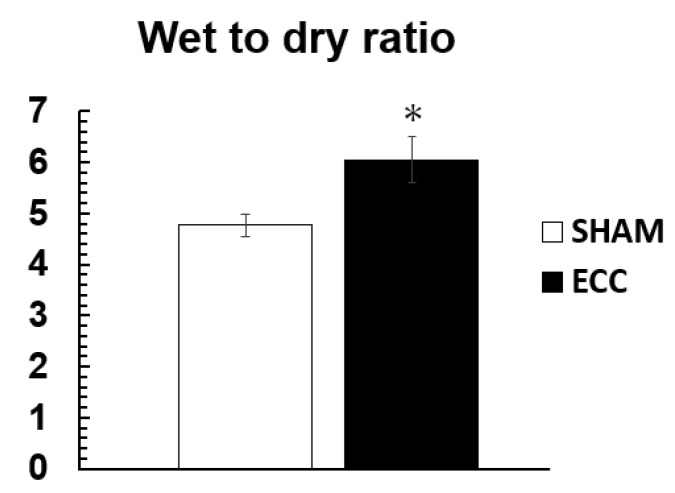
Wet-to-dry ratio of the left lung at the end of extracorporeal circulation. * *p* < 0.05 versus SHAM group.

The ECC group showed a significantly higher W/D ratio of the lung than the SHAM group (SHAM: 4.76 ± 0.21; ECC: 6.05 ± 0.46) (Figure 4).

## 4. Discussion

Our small animal ECC system was able to maintain blood gases, Hb, and blood pressure at appropriate levels. Furthermore, this model has the advantage of a low priming volume that does not require blood transfusion in ECC group rats. In our previous study, we showed that systemic inflammatory reactions and organ damage (including pulmonary edema) were induced in a rat ECC model [6]. While we were able to assess systemic inflammatory responses, we were not able to assess inflammation at the local organ level. In this study, we evaluated the inflammatory response at the local level in major organs using a real-time PCR assay. In addition, we observed that the pulmonary W/D was statistically significantly increased in the ECC group compared to the SHAM group, suggesting pulmonary edema and inflammation. ECC caused a significant localized organ inflammatory response. In particular, IL-6 was significantly increased in all major organs and plays a central role in circulation in vivo. In this study, the results support many previous studies showing that ECC induces a systemic inflammatory response and organ damage and stimulates the production of cytokines [3,7,8]. The reason for the marked increase in MCP-1 and IL-6 levels in the lungs is that ECC depletes venous blood from the right atrium and sends it to the common carotid artery, subsequently bypassing the animal’s own lung. We believe that this results in a marked decrease in pulmonary blood flow. Non-physiological organ blood flow in extracorporeal circulation may contribute to the promotion of inflammation. Accordingly, it is known that a decrease in bronchial artery blood flow occurs due to extracorporeal circulation, which, in turn, causes lung injury [4]. In addition, ischemia and reperfusion inhibit the production of nitric oxide (NO) from vascular endothelial cells and promote inflammation [9]. Non-physiological blood flow distribution by ECC is likely to cause local ischemic reperfusion in organs. Similarly, in this study, it is possible that ECC inhibited the production of NO in the lung capillaries and caused inflammation. In the future, we plan to focus on organ blood flow fluctuations during ECC.

Additionally, focusing on the inflammatory response cascade, MCP-1 is a monocyte chemotactic factor and has been shown to activate nuclear factor-κB (NF-κB), a transcription factor involved in inflammatory responses, by mobilizing regulatory and effector leukocytes [10]. Decreased blood flow and a slow velocity cause complex inflammatory reactions due to the activation of NF-κB in addition to the above-mentioned increase in NO due to vascular endothelial damage [11]. Additionally, it is known that the activation of NF-κB signaling is involved in the MCP-1-mediated induction of IL-6 [12]. Consistent with the hypothesis, this reduced pulmonary blood flow causes lung tissue damage and triggers NF-κB activation. Increased levels of cytokines such as interleukins exacerbate the inflammatory response. Accordingly, ischemia and reperfusion and lung transplantation are important factors. Ischemia and reperfusion with lung transplantation result in increased vascular permeability, decreased levels of alveolar macrophages, and the increased expression of inflammatory cytokines such as IL-6 (as shown in previous studies) [13]. Additionally, these complex interactions during ECC lead to further inflammation. We plan to continue our research focusing on the effects of ischemia and reperfusion during ECC. On the other hand, the levels of TNF-α, a tumor necrosis factor, were lower in the ECC group, although there was no statistically significant difference between the two groups. The reason for this may be that the half-life of TNF-α is very short (20 min), so the peak of gene expression may not have been captured in both groups. However, the time course of peak TNF-α gene expression is also prolonged when there is a sustained major invasion with necrosis [14]. In the activation of NF-κB signaling, TNF-α also induces the activation and increases the levels of MCP-1 and IL-6 in some pathways [15]. However, TNF-α is involved in the induction of NF-κB and cell survival responses [14]. In the ECC model of this experiment, wherein blood gases, Hb, and mean arterial pressure were maintained at appropriate levels, the apoptotic response progressed slowly, and the cellular environment was not conducive to a significant increase in TNF-α. In this study, the levels of MCP-1 and IL-6, but not TNF-α, were increased among the levels of inflammatory cytokines, suggesting that the inflammation caused by ECC was not derived from TNF-α. These findings suggest that if a body is well managed during ECC, organ injury will remain an inflammatory response, and apoptosis will not progress. It is thought that the appropriate control of blood pressure and Hb during ECC leads to the suppression of increased TNF-α.

In our previous study, we observed a systemic inflammatory response during and after blood tests assessing ECC, while the damage per organ localization caused by ECC was not examined. The novelty of this study is that it reveals that the inflammatory response caused by ECC is not uniform from organ to organ. We will continue our study to elucidate the pathophysiological mechanism of ECC. This miniature ECC model could be a very useful approach for basic research on pathophysiological mechanisms and ECC devices not only during ECC but also after ECC withdrawal.

Our study had several limitations. First, we evaluated parameters during short-term ECC. Further studies are needed to assess the systemic inflammatory response and organ damage during long-term ECC and after weaning ECC. Second, this study was conducted on healthy rats, and we have not been able to evaluate the studied parameters in a model with pathological conditions. In the future, we would like to conduct research focusing on the pathological conditions of diabetes and myocardial infarction, which have high incidence rates.

## 5. Conclusions

In this study, we examined the local inflammation of organs caused by ECC. We observed that the degree of gene expression of inflammatory cytokines differs depending on the organ. The levels of IL-6 observed were dependent on the organ and were especially increased in the heart and lungs.

## Figures and Tables

**Table 1:** Hemodynamic changes, Hb, partial pressure of blood gas, and electrolyte levels before and during ECC.

	Group	Pre-ECC	ECC 60 min	ECC 120 min
PaO_2_ (mmHg)	SHAM	116 ± 5	102 ± 8	107 ± 8
	ECC	108 ± 5	331 ± 24 *	345 ± 21 *
PaCO_2_ (mmHg)	SHAM	38 ± 1	37 ± 2	36 ± 1
	ECC	41 ± 1	36 ± 1	35 ± 1
Hb (g/dL)	SHAM	15.4 ± 1.3	15.2 ± 0.6	14.4 ± 0.5
	ECC	15.2 ± 0.3	10.0 ± 1.0 *	9.8 ± 0.8 *
pH	SHAM	7.38 ± 0.03	7.39 ± 0.03	7.40 ± 0.02
	ECC	7.39 ± 0.01	7.42 ± 0.02	7.39 ± 0.01
Na (mEq/L)	SHAM	139.5 ± 1.0	139.0 ± 2.4	138.0 ± 1.6
	ECC	138.3 ± 1.7	139.5 ± 1.9	141.3 ± 1.9
K (mEq/L)	SHAM	4.0 ± 0.1	4.0 ± 0.1	3.8 ± 0.3
	ECC	4.0 ± 0.2	5.2 ± 0.3 *	6.0 ± 0.4 *
Cl (mEq/L)	SHAM	108.8 ± 0.9	109.3 ± 1.2	108.0 ± 0.7
	ECC	109.5 ± 1.0	108.8 ± 0.5	108.8 ± 0.9

Variables are expressed by mean ± standard error. * *p* < 0.05 versus SHAM group at the same time. PaO_2_: partial pressure of arterial oxygen; PaCO_2_: partial pressure of arterial carbon dioxide; Hb: hemoglobin; pH: power of hydrogen

## Data Availability

All raw data are available upon reasonable request.

## Abbreviations

ECC	Extracorporeal membrane oxygenation
PaO_2_	Arterial pressure of oxygen
PaCO_2_	Arterial pressure of carbon dioxide
Hb	Hemoglobin
MAP	Mean arterial pressure
MCP	Monocyte Chemoattractant Protein
TNF-α	Tumor necrosis factor-α
IL	Interleukin
NF	Nuclear factor
W/D	Wet-to-dry
